# An Explainable Disease-Aware Intelligent Histopathological Representation Learning Framework for Generalizable Breast Cancer Classification

**DOI:** 10.3390/diagnostics16203264

**Published:** 2026-10-09

**Authors:** Faizan Ahmad, Irfanud Din, Khalil Khan

**Affiliations:** 1 Department of Computer Science and Information Technology, Superior University, Lahore 547700, Pakistan; fizanciit@gmail.com; 2 College of Information Technology Convergence, Gachon University, Seongnam 13120, Republic of Korea; irfan07@gachon.ac.kr; 3 Department of Information Technology, College of Computer, Qassim University, Buraydah 52571, Saudi Arabia

**Keywords:** disease-aware learning, histopathological representation learning, breast cancer classification, CNN, vision transformer, domain generalization, explainable artificial intelligence, deep learning, machine learning

## Abstract

**Background**: Deep learning approaches, especially convolutional neural networks (CNNs) and Vision Transformers (ViTs), have made substantial progress in breast cancer classification from histopathological images. Yet, previous CNN–ViT-based architectures frequently demonstrate insufficient generalization when evaluated on independent external datasets despite obtaining high performance on internal benchmarks. The key reason is that these architectures learn statistical dependencies instead of precisely identifying disease-specific morphological evidence. During optimization, models may unintentionally depend on non-diagnostic patterns, such as staining variations, scanner characteristics, acquisition settings, and dataset-specific textures, thereby potentially becoming aligned with class labels in the training data but not reflecting intrinsic pathological patterns. This phenomenon leads to inconsistent predictions under domain shifts. **Methods**: To overcome this limitation, this study proposes a disease-aware intelligent histopathological representation learning framework for generalizable breast cancer classification. The proposed framework leverages CNN and ViT encoders to capture complementary local cellular morphology and global tissue-level contextual information. A disease-aware representation learning mechanism is introduced to distinguish clinically relevant disease-specific features from non-diagnostic fluctuations, allowing the model to concentrate on transferable pathological features. Additionally, an intelligent adaptive decision module is designed to optimize the use of clinically relevant disease evidence for benign and malignant classification instead of relying on the conventional classification head. **Results**: The proposed framework is evaluated on BreakHis as the internal benchmark (98.10% accuracy) and various independent histopathology datasets for external validation. **Conclusions**: Comprehensive experiments, robustness analysis, and explainability analyses highlight the effectiveness of the proposed approach in learning generalizable disease-related representations and improving the consistency of cross-dataset breast cancer classification.

## 1. Introduction

Breast cancer is one of the most common and life-threatening diseases in the world, and there is a constant need for accurate, reliable, and early diagnostic strategies to enhance patient survival [1,2,3,4,5]. Histopathological image analysis is an essential part of breast cancer diagnosis because it provides detailed cellular morphology and tissue organization through microscopic examination [3,6,7,8]. Manual evaluation of histopathological slides is highly dependent on pathologist expertise and subject to inter-observer variability, fatigue, and diagnostic complexity, especially in difficult and complex cases. Thus, computer-aided diagnosis (CAD) systems using artificial intelligence have emerged as promising approaches for helping pathologists with objective and reproducible image-based analysis [4,6,9]. In recent years, deep learning has made great progress in histopathological image classification. Convolutional neural networks (CNNs) have shown a strong ability to learn local morphological patterns such as cellular structures, nuclear characteristics, and tissue textures. Vision Transformers use global self-attention mechanisms, enabling them to model long-range dependencies and large-scale tissue-level relationships. As a result, hybrid CNN–ViT architectures have received considerable attention because they integrate both local and global representation learning [2,5,10,11,12,13,14,15]. Although these methods have greatly improved performance on benchmark datasets, they still tend to show a significant drop in performance when tested on external datasets from various clinical settings. The primary problem is that existing deep learning models tend to learn statistical relationships in the training data instead of actual disease-specific pathological evidence. Histopathological images contain clinically relevant information and non-diagnostic variations. Disease-related patterns encompass abnormal cellular morphology, nuclear changes, variations in tissue architecture, and tumor-associated patterns. In contrast, non-diagnostic factors include staining variations, scanner-dependent appearance, acquisition protocols, laboratory preparation differences, and dataset-specific visual characteristics [6,8,12,16,17,18]. Previous CNN, ViT, and hybrid frameworks may unintentionally exploit these non-diagnostic patterns because they are often correlated with class labels in the training dataset. Consequently, models can achieve strong performance on internal datasets and fail to generalize when these irrelevant patterns change in unseen datasets [7,10,13,19,20,21,22,23]. While some methods (such as data augmentation, stain normalization, transfer learning, and domain adaptation) have been investigated to improve robustness, these methods generally focus on minimizing distribution differences and do not explicitly aim to separate disease-related information from unwanted variations [21,24,25,26]. Similarly, the traditional feature fusion approaches combine the CNN and ViT representations with no consideration of whether the features are derived from pathological evidence or from the characteristics of the datasets [1,13,17,19,23,27,28,29]. Hence, an effective disease-aware learning mechanism that can identify, preserve, and prioritize clinically relevant histopathological representations while suppressing the non-diagnostic information represents a critical research gap. Below Figure 1 shows the sample of internal BreakHis dataset.

Based on this motivation, this research investigates the following research questions:

RQ1: What is the reason behind the performance drop of the current CNN- and ViT-based histopathological classification models on external datasets despite high accuracy on internal benchmarks?

RQ2: Can a disease-aware representation learning strategy be effective to distinguish disease-specific pathological features from non-diagnostic image variations?

RQ3: Can prioritizing reliable disease-related features enhance cross-dataset generalization and classification reliability?

In order to answer these questions, this study proposes a disease-aware intelligent histopathological representation learning framework for generalizable breast cancer classification. The proposed framework utilizes CNN and ViT encoders to extract complementary information at the local (cellular) and global (tissue) levels [27,29,30,31,32,33,34,35]. Recent advances in digital pathology and artificial intelligence have expanded the use of computational methods for automated analysis across pathology and cytology applications [36,37]. These developments highlight the rising potential of digital solutions and AI-assisted analysis to enhance diagnostic support and handle complex pathological image data [38]. However, robust generalization across heterogeneous datasets remains an important challenge, motivating the development of disease-aware representation learning approaches for reliable histopathological classification [36,39].

In contrast to the traditional hybrid methods, which directly combine the extracted features, the proposed approach aims at learning the disease-aware representations by removing the irrelevant variations from the pathological evidence. In addition, an intelligent adaptive decision module is added to make use of reliable disease-specific information for final benign and malignant classification. The major findings of this study are outlined below:

1. To overcome the drawback of the existing deep learning models, which merge the disease-specific patterns with the non-diagnostic variations, a disease-aware intelligent representation learning framework is proposed.
2. A dual representation strategy is developed to learn and differentiate the clinically relevant pathological features from dataset-dependent information extracted through CNN and ViT encoders.
3. An adaptive intelligent diagnostic decision mechanism is introduced to prioritize reliable disease evidence rather than relying on a conventional classification head.

Extensive evaluation is performed on an internal benchmark dataset and multiple independent external histopathology datasets without retraining to assess the robustness, generalization ability, and reliability of the proposed framework. This research strives to transcend the traditional feature extraction and fusion methods and introduce a more biologically meaningful learning strategy to help deep learning models learn more transferable disease-specific representations for robust breast cancer histopathological image classification.

## 2. Literature Review

Deep learning has revolutionized breast histopathological image analysis by allowing the automatic extraction of complex visual features from microscopic tissue images. The early computer-aided diagnosis systems primarily depended on handcrafted features such as texture descriptors, morphological measurements, and statistical characteristics [1,3,5,6,7,8,9]. Although these methods were useful, they were limited in performance as hand-designed features were rule-based and did not adequately represent the complex and heterogeneous nature of cancer morphology. With the advent of deep learning, the classification of breast cancer has been greatly improved by automatically learning discriminative representations directly from images [8,12,15,16,17,18,19]. Convolutional neural networks (CNNs) are one of the most popular deep learning techniques used for histopathological image classification because of their excellent capability to capture local spatial patterns. To learn hierarchical features from low-level edges and textures to high-level cellular structures, several CNN architectures, such as VGG, ResNet, DenseNet, and EfficientNet, have shown promising results. CNN models have proven effective in breast histopathology by detecting key features such as nuclear irregularity, tissue texture, and cellular organization [22,27,35,40,41,42]. However, CNNs mainly use local receptive fields, which limits the model’s ability to capture long-range dependencies and wider tissue-level relationships that can be crucial in the accurate pathological interpretation. Recently, Vision Transformers (ViTs) have been introduced to overcome the limitations of CNN-based approaches for medical image analysis. In contrast to CNNs, ViTs rely on self-attention mechanisms to model the relations among distant regions of the histopathology image and to capture global contextual information. Transformer-based approaches have demonstrated better representation capabilities in histopathological classification tasks by learning the complex interactions between different regions of tissues. However, ViTs are typically large models that need large-scale training datasets and may not possess the same strong inductive bias as CNNs to capture the fine-grained cellular patterns, which may impact their effectiveness in limited medical imaging datasets [15,21,29,43,44,45].

Numerous recent studies have concentrated on hybrid CNN–Transformer architectures, utilizing the complementary properties of CNNs and ViTs. These methods are based on CNN-based local feature extraction and transformer-based global context modeling to ensure more comprehensive representation of images. Hybrid models show better classification results than the individual CNN or transformer models. Despite their good internal performance, many current hybrid methods are still problematic when validated on external datasets acquired under different clinical conditions. One of the main drawbacks of existing CNN, ViT, and hybrid models is their reliance on statistical patterns specific to the dataset. Histopathological images have both disease-related information and non-diagnostic variations where disease-related characteristics include abnormal morphology of cells, changes in nuclei, variation in tissue architecture, and tumor-specific patterns [18,26,31]. On the other hand, staining procedures, scanner settings, laboratory protocols, image acquisition conditions, and dataset characteristics can vary but do not represent disease biology but may still influence model decisions. Many existing deep learning models maximize the classification performance by utilizing any discriminative pattern in training data, which may result in shortcut learning. Consequently, models may attain superior performance on internal datasets while demonstrating reduced reliability on unseen external datasets. Various methods have been analyzed to enhance the model robustness against domain variation [30,35]. Data augmentation techniques try to improve diversity by creating transformed training samples while stain normalization techniques aim to reduce color differences among datasets. To enhance the feature extraction ability, transfer learning from large-scale natural image datasets has been extensively utilized. Moreover, to mitigate the distribution gap between the source and target domains, domain adaptation and domain generalization techniques have been introduced. However, these methods primarily aim at aligning the distribution of features and do not explicitly identify whether the learned representations are related to meaningful pathological evidence or irrelevant information specific to the dataset [41,42,43,44,45,46]. Recent advances in computational breast pathology have increasingly shifted from conventional task-specific convolutional neural networks toward transformer-based, self-supervised, and foundation-model approaches [47,48,49]. Recent review articles show that CNN and transformer architectures have been extensively explored for breast histopathology classification, segmentation, and diagnosis, and that challenges of limited datasets, staining variations, interpretability, and cross-dataset generalization persist [50,51]. Self-supervised learning (SSL) has been proposed to reduce reliance on large-scale, manually annotated data and learn transferable representations from unlabeled histopathological images. Recent pathology foundation models have been shown to learn generalizable representations from millions of unlabeled histopathological images, such as BEPH, pre-trained on 11 million unlabeled images and adapted to a series of cancer-related tasks. Similarly, recent benchmarking efforts have compared several pathology foundation models across different patient cohorts and found that generalization performance varies depending on the downstream task and data regimes. Together, these findings suggest that effective breast cancer image analysis should not only achieve strong predictive performance but also be carefully evaluated on heterogeneous datasets [52]. In this context, the present study contributes to recent research on foundation models and transformer-based models by proposing a multi-dataset breast cancer classification framework with patient-level separation with independent external evaluation, using a relatively task-specific and computationally practical architecture, while explicitly limiting its scope to classification [50,52]. To enhance robustness across heterogeneous histopathology datasets, recent computational pathology studies have investigated domain generalization, stain-invariant learning, self-supervised learning, and pathology foundation models. Domain generalization and stain-invariant methods mainly address distribution and staining variations, while self-supervised and foundation-model methods aim to learn transferable representations from large-scale data. However, these methods do not distinguish between disease-specific pathological information and dataset-dependent non-diagnostic variations. By contrast, the proposed framework uses a disease-aware representation strategy that highlights clinically relevant features while attenuating unwanted variations, improving cross-dataset generalization [36,49,51,52,53].

In recent years, to enhance the interpretability and reliability of deep learning models, attention mechanisms and feature selection strategies have been investigated. While these methods can identify salient image areas, they typically do not offer an explicit way to distinguish representations of disease from non-diagnostic variations [1,5,7,9]. Similarly, previous feature fusion frameworks integrate multiple representations but assume that all captured features contribute positively to classification, without evaluating their biological relevance [43,46,47,48]. Hence, there is a major research gap in designing disease-aware representation learning frameworks that can separate clinically meaningful pathological information from unwanted variations. In addition to high accuracy on internal datasets, a strong breast histopathology classification system should be able to learn disease-specific representations that can be transferred to other datasets and clinical settings. This study aims to overcome this limitation by proposing a Disease-Aware Intelligent Histopathological Representation Learning framework. The proposed framework aims to learn and select the disease-specific representations and minimize the reliance on non-diagnostic patterns, unlike conventional CNN, ViT, and hybrid methods that directly learn and combine features. The proposed method combines complementary CNN and ViT representations and introduces an intelligent disease-aware learning strategy that enhances the robustness, generalization, and reliability of breast cancer histopathology image classification. Table 1 shows the summary of limitations in breast cancer histopathology image classification models.

## 3. Methodology

### 3.1. Dataset Description

The proposed framework was trained and tested internally on the BreakHis breast histopathology dataset and externally validated on five independent datasets: BACH, Invasive Ductal Carcinoma (IDC), BRACS, BreCaHAD and TCGA-BRCA without retraining or fine-tuning. The BreakHis dataset was chosen as the main evaluation dataset, because it contains a variety of hematoxylin and eosin (H&E)-stained microscopic breast tissue images captured at various magnifications. It comprises 7909 RGB histopathological images from 82 patients, at 40×, 100×, 200× and 400× magnifications. The dataset offers significant variation in tissue morphology and imaging scale, which can be used to assess the ability of deep learning models to discriminate between benign and malignant breast lesions. The model was also tested on heterogeneous breast histopathology datasets from various sources and acquisition environments to assess the model’s generalization capabilities for external validation. The BACH dataset comprises H&E-stained breast microscopy images from the Grand Challenge on Breast Cancer Histology, and the IDC dataset comprises patches of breast histopathology images with annotations of the invasive ductal carcinoma regions from whole-slide images. The BRACS dataset consists of a large collection of breast carcinoma whole slide images and ROI annotations with various pathological patterns. In addition, BreCaHAD provides annotated breast histopathology images for different diagnostic tissue structures, while TCGA-BRCA provides a large-scale collection of breast cancer pathology images and clinical and molecular data. To ensure consistency across datasets, we formulated all experiments in this study as a binary classification problem with two classes: Benign and Malignant. We harmonized the original diagnostic categories in each dataset according to their pathological definitions. For BACH, Benign comprised Normal and Benign samples, and Malignant comprised In Situ Carcinoma and Invasive Carcinoma. For the IDC dataset, IDC-negative patches were used as the benign/non-IDC reference class and IDC-positive patches as the malignant/IDC class for the study-specific binary evaluation and details are shown in Table 2 below. Additionally, BCSS was used for quantitative XAI validation using expert-annotated pixel-level tissue masks. These masks served as reference regions for evaluating Grad-CAM, Grad-CAM++, and Score-CAM localization.

### 3.2. Datasets Preprocessing

These datasets were chosen to represent a wide range of patient cohorts, staining variations, imaging resolutions, magnifications, and pathological characteristics to allow for a comprehensive assessment. This multi-dataset evaluation approach can help to reliably estimate the robustness and generalization capabilities of the proposed framework beyond the training distribution. A standardized preprocessing pipeline was used prior to model training and evaluation to maintain consistent evaluation across the various breast histopathology datasets. All the labels were reduced to a binary classification problem with benign and malignant classes, as the original datasets have multiple diagnostic classes. This conversion was performed to make them consistent with the goal of this study, which is to automate the discrimination between non-cancerous (benign) and cancerous (malignant) breast tissue patterns. If there were several pathological subtypes in the dataset, benign-related categories were classified as benign, whereas invasive and carcinoma-related categories were classified as malignant. The binary formulation allows for clinically relevant analysis by directly addressing the primary question in breast cancer screening, namely the distinction between a malignant and a benign abnormality, as shown in Table 2. After label harmonization, all histopathology images were preprocessed with a consistent preprocessing pipeline. To be compatible with the deep learning architectures adopted, images were first resized to 224 × 224 pixels. To minimize variations due to staining and acquisition conditions, pixel intensity normalization was used to reduce the staining variation among slides and between different laboratories that is often found in histopathology images. Noise reduction methods like Non-Local Means (NLM) were also used to reduce irrelevant artifacts and to maintain important morphological structures as shown in Figure 2. These preprocessing procedures increase the consistency of the images while making it easier for deep learning models to learn discriminative pathological features. To avoid data leakage between training and testing subsets, a patient-wise split was used for the internal evaluation on the BreakHis dataset. The dataset was split into 70% training, 15% validation, and 15% test as shown in Table 3. BreakHis was strictly split at the patient level, with no patient overlap between training, validation, and test sets. External datasets were used only for independent testing and were not used for retraining or fine-tuning. During the training of the models, data augmentation was only performed on the training set, following an on-the-fly augmentation strategy after patient-level splitting. This method dynamically creates different variations of images without adding to the validation and testing data, which not only enhanced the generalization ability of the model but also ensured unbiased model performance. Moreover, five-fold cross-validation on the training set was performed to check the stability of the model and to obtain reliable performance estimates. The independent test set was used for internal evaluation of the final model, and external datasets were used solely for cross-dataset validation. Data augmentation was applied only to the training set after patient-wise splitting using an on-the-fly strategy, where random transformations were generated dynamically during training without storing additional images. The applied augmentations included random rotation, horizontal and vertical flipping, translation, zooming, brightness adjustment, and color jitter. Class balancing was applied only during training using on-the-fly augmentation, while validation and testing sets remained unchanged.

### 3.3. Proposed Disease-Aware Intelligent Representation Learning Framework

The proposed framework is based on a progressive evaluation strategy that involves two key stages: baseline representation analysis and disease-aware feature learning. First, multiple cutting-edge deep learning architectures are explored on the internal BreakHis dataset in order to determine their individual representation capabilities. The goal of this stage is not only to identify the baseline performance but also to find the most appropriate CNN and Vision Transformer backbones to build the proposed framework. The first phase involves evaluation of the baseline models and optimal backbone selection. The BreakHis dataset is chosen as the internal benchmark for the development and evaluation of the proposed framework because it contains a variety of H&E-stained breast histopathology images with considerable variations in tissue morphology and magnification. To prevent data leakage, the dataset is split into training (70:15:15), validation, and testing sets using a patient-independent approach. First, four deep learning models (representative) are trained separately using the BreakHis training set:

1. The conventional CNN architecture, VGG16, is used to extract g hierarchical local visual patterns.
2. ResNet50 is an advanced CNN architecture with residual feature connectivity.
3. As a transformer-based model, Vision Transformer (ViT-Base/16) can learn global contextual relationships.
4. Swin Transformer is hierarchical transformer architecture for modeling multi-scale spatial dependencies.

These models represent two complementary learning paradigms. CNN-based models tend to be dominated by local pathological features, such as cellular structures, texture variations, and morphological patterns, while transformer-based models capture the global relationships between different tissue regions. Every baseline model is optimized separately and assessed by accuracy, precision, recall, F1 score, and AUC. The goal is to find the best feature extractor in each architectural group and not to directly fuse all models. Based on the overall classification capability and the quality of feature representation, the best-performing CNN and ViT models are selected. Mathematically, the evaluated model set is defined as:

(1) $$M=\{{CNN}_{1},{CNN}_{2},{ViT}_{1},{ViT}_{2}\}$$

(2) S=αAcc+βF1+γAUC

(3) $$C^{*},V^{*}=arg\;Max(S_{M})$$

The performance score is calculated as in Equation (2), while $C^{*}and\;V^{*}$ represent the selected CNN and ViT feature extractors in Equation (3). The feature representations of CNN and Vision Transformer are inherently heterogeneous and can be combined by hybrid CNN–Vision Transformer architecture, but such a combination is actually a fusion of local morphological patterns and global contextual information. CNNs mainly capture localized spatial characteristics such as cellular texture and structural variations, whereas Vision Transformers learn long-range dependencies through self-attention mechanisms. These features can be directly combined, but this can lead to dimensional inconsistencies and distributional differences, which can adversely impact the subsequent feature learning process. Hence, we propose a Cross-Representation Feature Alignment Module (FAM) to align CNN and transformer embeddings into a shared latent representation space prior to disease-aware learning. Given an input histopathological image:

(4) $$X\in R^{H\times W\times C}$$

The image is processed through parallel CNN and Vision Transformer encoders. VGG16 is used to capture the local pathological features, while Swin Transformer extracts the global tissue-level dependencies. The extracted feature representations are formulated as:

(5) $$Z_{c}=f_{CNN}(X;\theta_{c})$$

(6) $$Z_{v}=f_{ViT}(X;\theta_{v})$$

where $Z_{c}$ and $Z_{v}$ show the CNN and transformer feature embeddings, respectively, while $\theta_{c}$ and $\theta_{v}$ show their learnable parameters. Since both feature vectors have different dimensions and statistical distributions, FAM applies learnable projection layers to attain feature compatibility:

(7) $$P_{c}=W_{c}Z_{c}+b_{c}$$

(8) $$P_{v}=W_{v}Z_{v}+b_{v}$$

where $W_{c}$ and $W_{v}$ are the trainable projection matrices, while $b_{c}$ and $b_{v}$ show the bias terms. Such transformation vectors both highlight shared latent spaces and prevent their discriminative characteristics. The aligned feature representation is then obtained through adaptive additive fusion:

(9) $$Z_{a}=P_{c}+P_{v}$$

where $Z_{a}$ shows the unified feature embedding. Unlike direct feature fusion, this compact alignment technique minimizes the redundancy of features and prevents dominance of one representation type. The resulting vector combines the CNN-based local morphological evidence with transformer-based global contextual information. In summary, FAM (supporting module) offers the initial harmonization level of the proposed framework by creating a compatible feature vector for subsequent disease-aware representation learning. However, FAM is not considered the key contribution; it allows reliable interaction among heterogeneous CNN and transformer features and enhances the reliability of downstream diagnostic modules.

Even though FAM offers a consistent CNN–Transformer representation, there might be irrelevant variations within the aligned feature space due to staining, scanner, illumination, and acquisition conditions, in addition to disease-related patterns. In conventional hybrid architectures, all of extracted features are fused equally, but it is possible that some of these features are not diagnostically relevant and may affect the classification process. Hence, we propose a Disease-Aware Feature Separation Module (DAFSM) to explicitly separate the diagnostic information from nuisance variations and learn a more robust disease-specific representation. The aligned feature representation that is output from FAM is the input to DAFSM:

(10) $$Z_{a}\in R^{d}$$

where $Z_{a}$ is the unified CNN–Transformer embedding. To estimate the contribution of each feature dimension towards disease discrimination, DAFSM introduces a learnable disease-aware gating mechanism:

(11) $$G_{d}=\sigma (W_{g}Z_{a}+b_{g})$$

where $G_{d}$ is the disease relevance weights, $W_{g}$ and $b_{g}$ are trainable parameters, σ(.) is the sigmoid activation function. The resulting gate values reflect the relevance of the individual feature elements for the recognition of pathological features. With the learned disease relevance weights, the disease-specific representation is:

(12) $$Z_{d}=G_{d}⊙Z_{a}$$

Here, $Z_{d}$ is the extracted disease-aware representation and ⊙ is the element-wise multiplication. The operation boosts discriminative pathological characteristics and diminishes less relevant responses. The complementary nuisance representation is given as:

(13) $$Z_{n}={(1-G}_{d})⊙Z_{a}$$

where $Z_{n}$ is non-diagnostic information like imaging variations and acquisition effects. By explicitly modelling this component, the classifier is not allowed to use irrelevant patterns. A disentanglement constraint is used to encourage good separation between disease-specific and nuisance features:

(14) $$L_{sep}=||Z_{d}^{T}Z_{n}{\left|\right|}_{2}$$

where $L_{sep}$ minimizes the dependency between both representations. This constraint helps the disease branch to maintain clinically meaningful information and to minimize redundant and irrelevant feature interactions. In summary, DAFSM is an explicit disease-aware feature disentanglement mechanism to distinguish the proposed framework from the traditional CNN–Transformer fusion. DAFSM filters out nuisance variations, while preserving pathological evidence, to produce a more powerful representation that helps to increase the generalization ability of the network in different histopathological imaging conditions. The proposed Disease Evidence Attention Module (DEAM) further refines the disease-aware representation.

DAFSM distinguishes between the characteristics of the disease and nuisance variations, but the resulting disease representation can contain characteristics of varying diagnostic value. Some morphological characteristics, including the arrangement of cells, irregularities in cell nuclei, and irregularities in the structure of tissue, are more indicative of malignancy in histopathological images than others. Thus, an adaptive refinement mechanism is needed to emphasize the most informative disease-related features. To overcome this, the second novel module of the framework, called Disease Evidence Attention Module (DEAM), is proposed. DEAM is a method to refine the disease-specific features by assigning adaptive importance weights to the separated disease- specific representation from DAFSM. DEAM is a novel attention mechanism that is different from standard attention mechanisms used on generic feature embeddings; it is applied after disease-aware separation, which allows the model to selectively boost clinically meaningful pathological evidence.

(15) $$Z_{d}\in R^{d}$$

$Z_{d}$ is the embedding of the disease-specific feature obtained by DAFSM. The module first ranks the importance of the features with a learnable attention mechanism:

(16) $$A=Softmax(W_{a}Z_{d})$$

where A denotes adaptive attention weights and $W_{a}$ denotes the learnable attention projection matrix. To enable the network to assign a higher weight to highly discriminative pathological features, the feature responses are normalized using the SoftMax operation. The attention-guided disease evidence representation is created as:

(17) $$Z_{e}=A⊙Z_{d}$$

$Z_{e}$ Represents the refined disease-evidence representation, and ⊙ is the element-wise multiplication. This operation enhances key diagnostic characteristics and decreases the impact of less informative responses. To preserve the original disease representation and avoid excessive feature suppression, a residual refinement strategy is applied:

(18) $$Z_{enh}=Z_{d}+Z_{e}$$

where $Z_{enh}$ is the final refined disease representation? The residual connection preserves key disease information and integrates pathological evidence guided by attention. In summary, DEAM offers an adaptive disease-evidence refinement mechanism, highlighting the most informative features in the disease-specific representation. The attention operation is carried out following the feature disentanglement, so that the module focuses only on clinically relevant information but not background variations. The improved representation is then used by the Adaptive Intelligent Decision Module (AIDM) to make the final diagnostic decision.

The last step of the proposed framework is to transform the refined disease representation to the final diagnostic prediction. FAM, DAFSM, and DEAM gradually enhance the quality of features by aligning and separating them and by refining the evidence, but there is still a possibility that the reliability of the extracted disease-related information may differ from one histopathological sample to another. Conventional classification heads are usually based on the fixed fully connected layer and assume that all learned features are equally important in the final decision. However, in ambiguous cases where benign and malignant patterns overlap, an adaptive decision mechanism, based on feature reliability, is required. To overcome this limitation, the proposed Adaptive Intelligent Decision Module (AIDM) is introduced as the last novel module of the framework. AIDM is confidence-guided diagnostic inference mechanism which estimates reliability of the improved disease representation prior to classification. The module adaptively weights the pathological evidence, which is reliable, and minimizes the weight of the uncertain feature responses to avoid directly mapping features to predictions. Unlike a conventional fully connected classification head, AIDM introduces a learnable adaptive confidence generation mechanism that dynamically recalibrates enhanced disease representations before final prediction. The input representation of AIDM is obtained from DEAM:

(19) $$Z_{enh}\in R^{d}$$

where $Z_{enh}$ is the enhanced disease representation with refined pathological evidence. AIDM first computes the confidence level of each feature component by a learnable confidence mechanism:

(20) $$C=\sigma (W_{c}Z_{enh}+b_{c})$$

where (C) is the confidence vector, $W_{c}$ and $b_{c}$ are learnable parameters, and σ(.) is the sigmoid activation function. The confidence scores generated show the reliability of the individual feature responses for the diagnostic decision-making. The confidence- weighted representation is then computed as:

(21) $$Z_{c}=C⊙Z_{enh}$$

where $Z_{c}$ is the adapted feature representation and ⊙ is the element-wise multiplication. This operation enhances the weight of highly reliable disease evidence, while dampening uncertain responses. The final prediction probability is calculated by a classification layer:

(22) $$\hat{y}=Softmax(W_{p}Z_{c}+b_{p})$$

where $\hat{y}$ is the predicted class probability and $W_{p}$ and $b_{p}$ are parameters of the classification. The model is trained with categorical cross-entropy loss:

(23) $$L_{cls}=-\sum_{i=1}^{N}y_{i}log({\hat{y}}_{i})$$

where $y_{i}$ is the true label and ${\hat{y}}_{i}$ is the predicted probability. In short, AIDM is an adaptive confidence-guided decision strategy that goes beyond traditional static classification heads. AIDM, with feature reliability estimation prior to prediction, enhances the robustness of diagnostic decision, especially for the difficult histopathological samples in which the morphological characteristics are uncertain. The module transforms subtle pathological evidence to reliable benign and malignant classification results, which completes the proposed disease-aware representation learning framework. The proposed Disease-Aware Intelligent Representation Learning Framework includes one supporting feature alignment module followed by three novel components:

1. The Cross-Representation Feature Alignment Module FAM (Supportive Module);
2. The Disease-Aware Feature Separation Module (DAFSM);
3. The Disease Evidence Attention Module (DEAM).

The Adaptive Intelligent Decision Module (AIDM)

Among these, DAFSM, DEAM, and AIDM constitute the primary methodological contributions of the proposed framework, whereas FAM facilitates the alignment of heterogeneous CNN and Vision Transformer feature representations prior to disease-aware learning. Below Table 4 highlights the summary of the proposed disease-aware intelligent representation learning framework. The complete training procedure of the proposed framework is summarized in Algorithm 1.
**Algorithm 1:** Training Procedure of the Proposed Framework1.Initialize the VGG16 encoder, $f_{CNN}(.)$, and Swin Transformer encoder $f_{ViT}(.)$, Feature Alignment Module (FAM), Disease-Aware Feature Separation Module (DAFSM), Disease Evidence Attention Module (DEAM), and Adaptive Intelligent Decision Module (AIDM)2.For each training sample ${(X}_{i,}y_{i})$ ∈ D do3.Extract the local CNN representation$\;Z_{c}=f_{CNN}(X;\theta_{c})$4.Extract the global Transformer representation$\;Z_{v}=f_{ViT}(X;\theta_{v})$5.Align heterogeneous feature representations using FAM$\;Z_{a}$ $=\mathrm{FAM}(Z_{c}$ $,\;Z_{v})$6.Separate disease-specific and nuisance representations using DAFSM ${(Z}_{d}$ $,\;Z_{n})$ $=\mathrm{DAFSM}(Z_{a})$7.Enhance disease-related evidence using DEAM $Z_{enh}=DEAM(Z_{d})$8.Generate confidence-guided prediction using AIDM $\hat{y}$$\;=\;\mathrm{AIDM}\;(Z_{enh})$9.Compute classification loss $\;L_{cls}=CE($$\hat{y}$, $y_{i})$10.Compute disease separation loss$\;L_{sep}$11.Compute total optimization objective$\;L_{total}=L_{cls}+{\lambda L}_{sep}$12.Update all learnable parameters using backpropagation and the Adam optimizer13.End For14.Return

### 3.4. Experimental Setup

The proposed DAIRLF framework was developed and evaluated on the internal BreakHis dataset, while the BACH, IDC, BRACS, BreCaHAD, and TCGA-BRCA datasets were used for external validation to analyze the generalization ability of the proposed framework. All histopathological images were resized to 224 × 224 pixels with three channels before training. A strict patient-wise data split mechanism was applied in a 70-15-15 ratio, using five-fold cross-validation to prevent data leakage and ensure fair evaluation. Stain normalization and intensity normalization were implemented during preprocessing to minimize the color variations across histopathological slides. Data augmentation methods, such as random rotations, horizontal and vertical flipping, translation, zooming, brightness adjustment, and color jitter, were applied during training to enhance model robustness and reduce the risk of overfitting. The experimental framework was implemented in Python 3.11 using the PyTorch 2 deep learning library, while The implementation was developed using Scikit-learn 1.5.2, NumPy 1.26.4, Pandas 2.2.3, OpenCV 4.10.0, Pillow 10.4.0, and Matplotlib 3.9.2. were utilized for data preprocessing, performance evaluation, and visualization. Model training was conducted on a workstation equipped with an NVIDIA RTX 4090 GPU (24 GB VRAM), an Intel Core i9 processor, 128 GB RAM, Ubuntu 22.04 LTS, CUDA 12.x, and the corresponding cuDNN libraries. The whole network was optimized end-to-end using the AdamW optimizer with an initial learning rate of 1 × 10^−4^, a weight decay of 1 × 10^−5^, a batch size of 32, and a maximum of 100 epochs. A ReduceLROnPlateau scheduler and early stopping with a patience of 15 epochs were applied to improve convergence and avoid overfitting. The overall optimization objective integrates the cross-entropy classification loss with the proposed disease separation loss. Model performance was quantitatively assessed using Accuracy, Precision, Recall, Specificity, F1-score, AUC, and Cohen’s Kappa, while Grad-CAM, Grad-CAM++, and Score-CAM were employed to provide visual explanations of the diagnostic decisions and validate the interpretability of the proposed framework as shown in Table 5.

## 4. Experimental Results

### 4.1. Baseline Models Training and Evaluation

Identical preprocessing, augmentation, optimization, and evaluation settings are utilized for all models to ensure a fair comparison. Among all evaluated CNN and ViT architectures, VGG16 and the Swin Transformer achieved superior classification performance. VGG16 attained 93.40% accuracy and an F1-score of 93.10% on the internal BreakHis dataset, outperforming ResNet50 across all evaluation metrics as shown in Table 6. The reported enhancements highlight that VGG16 reliably extracted the fine-grained local morphological characteristics, while the hierarchical self-attention of the Swin Transformer offered richer global contextual representations for histopathological image analysis. Based on these experimental results, VGG16 and the Swin Transformer were chosen as the optimal CNN and Vision Transformer backbones, respectively, and further incorporated into the proposed Disease-Aware Intelligent Representation Learning Framework for feature extraction and downstream disease-aware representation learning.

### 4.2. Ablation Study of Proposed Components

To evaluate the contribution of each proposed module in the proposed framework, a comprehensive ablation study was conducted as shown in Table 7 while row-wise normalized confusion matrix are shown in Figure 3 below. The analysis evaluated the effects of nuisance feature suppression, Disease-Aware Intelligent Representation Learning Module (DAIRLM), and Adaptive Intelligent Decision Module (AIDM). Two major complementary components are used in the proposed DAIRLM: the Disease-Aware Feature Separation Module and the Disease Evidence Attention Module (DEAM). The DAFSM improves feature learning by separating essential disease-related patterns from less informative feature responses, On the other hand, DEAM further enhances the captured features by focusing on relevant and core disease evidence. The combination of both components enables the framework to learn a more reliable and focused disease-aware feature space. The FC–Softmax classifier achieved 96.95% accuracy in the initial ablation setup without any proposed enhancement. This configuration is based on the original CNN–Vision Transformer fusion without disease-aware refinement. Adding the proposed DAIRLM with nuisance feature suppression, which shows the impact of disease-aware feature separation and evidence enhancement, further improved the quality of learned representations by minimizing the influence of irrelevant feature and preserving diagnostically essential and meaningful patterns. However, the entire proposed system with the Adaptive Intelligent Decision Module (AIDM) achieved stable performance with 98.10% accuracy. The additional enhancement attained after incorporating the AIDM further validates that adaptive feature prioritization and refinement of the disease representation is achieved by giving higher priority to the most discriminative and essential features before final classification. Further, the improvements in F1-score, Kappa, MCC and AUC score indicate the reliability of the proposed modules for enhancing classification reliability. The consistent improvement in performance from DAIRLM to DAIRLM + AIDM confirms that each of the proposed modules is beneficial to the final decision-making process. The minimum standard deviation of the overall proposed model also illustrates better stability and robustness. To conclude, the ablation study results confirm that DAIRLM is able to learn disease-aware representations through feature separation and disease evidence improvement, and that AIDM provides adaptive decision-oriented feature refinement, which leads to stable benign–malignant classification performance.

The major aim of external validation is to examine the cross-dataset generalization ability of the proposed framework without retraining or fine-tuning on five independent breast histopathology datasets (BACH, IDC, BRACS, BreCaHAD, and TCGA-BRCA) to observe whether the proposed noise suppression approach can improve robustness across different data distributions and acquisition settings. The proposed framework was directly applied to external datasets, and only the noise suppression module was enabled and disabled to make a fair comparison. The experimental results show that performance on all external datasets is consistently improved with the addition of noise suppression. By using noise suppression, the proposed framework achieved 95.82–96.48% on the BACH dataset and similar results were reported on the remaining external datasets as shown in the following Table 8. The consistent improvements demonstrate that non-discriminative feature responses can be suppressed to reduce the effects of dataset-specific variations and achieve more transferable and reliable disease-specific representations. Furthermore, the steady improvement in F1 score, Kappa, and AUC confirms that noise suppression not only improves the classification accuracy but also enhances classification reliability. The strong performance on various external datasets demonstrates that the proposed framework is not dataset-specific but can effectively generalize to unseen breast histopathological images. In summary, the external validation results corroborate the proposed disease-aware representation learning approach with noise suppression to provide more stable and transferable feature representations for benign and malignant breast cancer classification.

The normalized confusion matrices were used to understand the class-wise prediction behavior of the proposed framework on the external validation datasets. The matrices show the distribution of correctly and incorrectly classified benign and malignant samples when applying the noise suppression setting. The larger diagonal values signify a good discriminate d ability of model on unseen datasets while the decreased off-diagonal errors signify better robustness and generalization ability. The comparison between noise suppression enabled and disabled settings further shows the contribution of noise-aware preprocessing in minimizing the misclassification errors. Below Table 9 shows the class-wise performance while Figure 4 and Figure 5 shows the confusion metric and Figure 6 demonstrate the roc-curve analysis of proposed framework on external datasets.

The ROC curves offer a threshold-independent evaluation of the proposed framework by showing the trade-off between true positive rate and false positive rate across external datasets. The consistently stronger AUC attained with noise suppression highlights better separability between benign and malignant classes. The proposed framework shows better ROC performance than the noise-suppression-disabled setting, which indicates better generalization ability when tested on the independent datasets without retraining. To further analyze the effectiveness of the proposed framework in learning discriminative disease-aware representations, a t-SNE-based feature visualization analysis was performed before and after incorporating the proposed modules. As shown in Figure 7 below, the baseline feature representations show high overlap between the benign and malignant samples, representing low class separability and irrelevant variations. The learned feature embeddings of the proposed framework are more compact within each class and show a high degree of separation between benign and malignant classes. This improvement suggests that the proposed modules can effectively refine features by removing non-informative variations and retaining clinically relevant pathological patterns. The better feature distribution also demonstrates the ability of the proposed framework to learn generalizable representations for breast cancer classification.

An external validation analysis was performed on five independent breast cancer histopathology datasets (BACH, IDC, BRACS, BreCaHAD and TCGA-BRCA) to assess the contribution of the noise suppression mechanism to cross-domain generalization. A detailed study of the effect of noise suppression on external robustness is shown in Figure 8. Figure 8a shows the accuracy drop from the internal BreakHis dataset to external datasets. In the absence of noise suppression, the framework is more likely to suffer a performance loss as a result of the differences in staining patterns, acquisition devices, tissue morphology, and dataset-specific characteristics. The noise-suppressed model, on the other hand, consistently reduces the performance degradation gap for all external datasets, which demonstrates better preservation of disease-relevant representations. The direct accuracy improvement that is obtained by the proposed noise suppression mechanism is shown in Figure 8b. The model achieves stable gains on all external datasets, ranging from 0.66% to 1.04%, demonstrating that suppressing irrelevant image variations leads to better transfer of features. The improvement is further confirmed by employing several evaluation metrics such as accuracy, F1-score, and AUC as shown in Figure 8c. The stable improvement in all metrics suggests that the noise suppression is beneficial for overall classification accuracy and also for class discrimination capability and prediction reliability. The domain shift sensitivity analysis is shown in Figure 8d where external accuracy varies across different datasets. A smaller variation shows more consistent performance across different data distributions. The framework with noise suppression shows less variation in accuracy than the baseline framework, indicating better robustness to external domain variations. In summary, these results validate that the proposed noise suppression module can effectively reduce the reliance on the dataset characteristics and enable the framework to learn more transferable disease-specific features, thereby enhancing the generalization ability of the framework on unseen histopathology datasets.

The representation probe analysis further validates the disease-aware disentanglement achieved by DAFSM as shown in Table 10. The disease representation Zd retains strong disease-related information while substantially reducing dataset-specific information, whereas the nuisance representation Zn shows the opposite behavior. This separation provides additional evidence that the proposed framework distinguishes diagnostic features from non-diagnostic variations rather than merely improving classification performance.

### 4.3. Statistical Analysis

To determine whether the performance gains of the proposed framework compared to the baseline models were statistically significant, statistical analyses were conducted with the five cross-validation folds as the experimental units. All statistical analyses were performed using fold-level accuracy values obtained from five-fold cross-validation. The mean and standard deviation (SD) were calculated for each model. Paired *t*-tests were performed to compare the proposed framework with each baseline model (ResNet50, VGG16, ViT-B/16, and Swin Transformer) on a pair wise basis. The Friedman test was used for the overall comparison of all five models. Cohen’s d was used to quantify the magnitude of pair wise differences, and 95% confidence intervals (CIs) were calculated for the mean accuracy differences. A significance level of *p* < 0.05 was used. These analyses were conducted at the fold level rather than on individual augmented images to avoid treating augmented samples as independent experimental observations. The full statistical results are given in Table 11 below.

The proposed framework achieved the highest accuracy across the evaluated models (98.10 ± 0.05%), compared with ResNet50 (92.84 ± 0.28%), VGG16 (93.40 ± 0.24%), ViT-B/16 (94.21 ± 0.22%), and Swin Transformer (94.70 ± 0.19%). The paired statistical analysis showed significant performance differences between the proposed framework and the baseline models. The Friedman test also showed that there was a significant difference between the evaluated models. The effect-size estimates and 95% confidence intervals are also included to gain further insight into the size and accuracy of these differences in performance.

### 4.4. Explainable Analysis

A controlled noise-based sensitivity analysis was utilized to analyze the robustness and reliability of the proposed explainable framework against image-level perturbations. Initially, a preprocessed histopathological image was loaded as input, and Gaussian noise was artificially applied to real-world image degradation due to acquisition setting and variation, sensor artifacts, and environmental disturbances as shown in Table 12. The noise perturbation was added using a zero-mean Gaussian distribution with a standard deviation of σ = 45, which offers a medium level of corruption while protecting the underlying tissue morphology. The noisy image was then processed via a noise suppression level to analyze whether the relevant diagnostic areas and attention patterns can be recovered after degradation. For every input (original, noisy, and noise-suppressed images), explainability analysis was implemented by Grad-CAM, Grad-CAM++, Score-CAM, and LIME. Our key objective was not only to validate the classification reliability and stability but also to analyze whether the model maintained its focus on disease-relevant morphological structures under different image perturbations. In the original image, the corresponding explanations highlight the focused attention on relevant histopathological areas linked with the target class. After adding noise (σ = 35), slight attention dispersion was noted, showing that random perturbations can add irrelevant activation areas and increase model uncertainty. Furthermore, with noise suppression, the attention maps became more localized and more reliable with the original diagnostic areas, showing the enhanced ability of the framework to protect meaningful and informative feature representations despite input degradation. Extensive robustness analysis shows that the proposed approach is not based on isolated pixel-level patterns; instead, it extracts the stable morphological features that remain informative under moderate image disturbances.

We quantitatively validated the explainability of the proposed CNN–ViT framework using Grad-CAM, Grad-CAM++, and Score-CAM against corresponding expert-annotated BCSS regions as shown in Figure 9 and Figure 10 below. We kept each selected BCSS case, its original histopathological image, and its ground-truth annotation as-is. We threshold the resulting XAI heatmaps into binary localization maps and compared them with the reference annotations. We used Dice Score, Intersection over Union (IoU), Precision, Recall, and Localization Accuracy to quantify spatial agreement between the model explanations and annotated pathological regions. Results are presented as Mean ± SD for the cases evaluated to assess localization performance and consistency. This analysis supports the qualitative XAI visualizations and provides quantitative evidence of whether the proposed framework focuses on relevant histopathological regions. We selected the three XAI techniques to provide complementary interpretations of the CNN–ViT predictions. While Grad-CAM offers relatively coarse localization of class-discriminative feature regions, Grad-CAM++ provides more localized activation around important regions, and Score-CAM estimates importance via activation-based scoring without directly relying on gradients. We quantitatively compared them using the same BCSS annotations and evaluation protocol. This comparison lets us evaluate spatial consistency between the highlighted regions and the annotated tissue regions, rather than relying on visual inspection alone.

## 5. Discussion

This study aimed to address the problem of poor cross-dataset generalization for the classification of breast histopathological images. While modern deep learning models, such as convolutional neural networks (CNNs), Vision Transformers (ViTs), and their hybrid models, have demonstrated high accuracy on benchmark datasets, their performance on external datasets is often lower. This restriction is primarily due to the fact that deep models learn statistical correlations from the training data to optimize the classification task. Therefore, models may extract not only disease-related morphological features but also non-diagnostic variations such as staining differences, scanner properties, acquisition protocols, and dataset-specific visual patterns. To overcome this limitation, we propose a disease-aware intelligent histopathological representation learning framework, which incorporates a representation learning strategy that aims to distinguish the disease-relevant information from irrelevant variations. Baseline analysis shows that CNN and ViT architectures are complementary. CNN-based models excel at capturing local pathological structures, such as cellular morphology, nuclear characteristics, and texture patterns, while ViT-based models excel at capturing global contextual information, modeling long-range dependencies among tissue regions. However, direct fusion of these architectures can result in both relevant and irrelevant information. The proposed disease-aware feature separation module overcomes this limitation by dynamically emphasizing the diagnostically meaningful representations. The robustness of the proposed framework is due to its ability to focus on transferable disease features rather than dataset-specific patterns. Comparison of the proposed framework with Conventional CNN–Transformer hybrid architectures is shown in Table 13 below.

The proposed approach incorporates adaptive feature weighting and disease evidence refinement, which differs from conventional hybrid models that assume that all extracted features have equal weights in the prediction. This allows the model to learn pathological features that are common to all datasets and imaging settings. To analyze the decision-making behavior of the proposed framework, an explainability analysis was performed using multiple XAI techniques, such as Grad-CAM, Grad-CAM++, Score-CAM, and LIME. The visualization results of Grad-CAM and Grad-CAM++ showed that the model concentrated more on the diagnostically relevant tissue regions rather than background information and irrelevant image areas. Score-CAM also validated the importance of salient activation regions in an activation-agnostic manner, which further increased confidence in the stability of the learned representations. To offer local explanations, LIME analysis identified regions of the image that contributed to a particular prediction. The consistency between various XAI methods indicates that the proposed model is learning meaningful pathological features related to breast cancer classification. The proposed framework is further validated through statistical evaluation. The fold-wise analysis shows similar performance across the five folds, minimizing the possibility that the improvements are due to random variation. Further evidence of the superiority and stability of the proposed method over the baseline methods is provided by statistical significance tests. Although the proposed framework achieved strong performance, some limitations remain. While several publicly available external datasets are utilized, histopathological datasets remain limited in clinical settings and may not be sufficient to reflect global variability. Future studies should include larger multicenter datasets and prospective clinical validation. Moreover, the current framework is based solely on image information and does not account for complementary clinical and molecular information. The possibility of multimodal disease-aware learning by combining histopathological images and clinical/genomic data can be explored in future studies. Overall, these results highlight that enhancing generalization requires moving beyond conventional feature extraction and fusion strategies toward learning biologically meaningful disease representations. Summary of performance comparison and limitations in breast cancer image classification models are shown in Table 14.

## 6. Conclusions

In this study, we introduce the Disease-Aware Intelligent Histopathological Representation Learning framework to classify breast cancer robustly and consistently from histopathological images. Previous CNN, Vision Transformer, and hybrid methods have yielded good performance on internal datasets; however, their performance on independent external datasets is frequently poor, as they can learn correlations between datasets rather than the actual disease-related features. To tackle this challenge, a disease-aware learning approach is proposed that distinguishes clinically significant pathological representations from non-diagnostic variations. The framework combines complementary CNN and Vision Transformer representations to capture both local cellular morphology and global tissue-level information. The proposed method uses disease-aware feature separation, disease evidence refinement, and adaptive intelligent decision-making to select reliable pathological information for final classification rather than directly fusing the extracted features. This design allows the model to address staining variability, acquisition differences, and other dataset-specific factors that are often encountered in deep learning-based histopathology systems. Extensive testing with an internal BreakHis benchmark dataset and multiple independent external histopathology datasets was performed to validate whether the proposed framework is effective in improving robustness and generalizability. Moreover, explainability analysis by Grad-CAM, Grad-CAM++, Score-CAM, and LIME demonstrates that the model concentrates on the meaningful pathological regions related to cancer diagnosis, thereby enhancing model transparency and interpretability. The proposed methodology is a step toward designing clinically reliable AI systems for analyzing breast cancer histopathology subject to further real-world validation, with a focus not only on feature extraction but also on disease-aware representation learning. However, further investigation using larger multicenter datasets, prospective clinical studies, and multimodal information sources are required to translate the proposed framework into practical clinical settings. Finally, this study emphasizes the need for transferable disease-specific representations and offers a valuable method to address the lack of generalization in existing deep learning methods for medical image analysis.

## Figures and Tables

**Figure 1:**
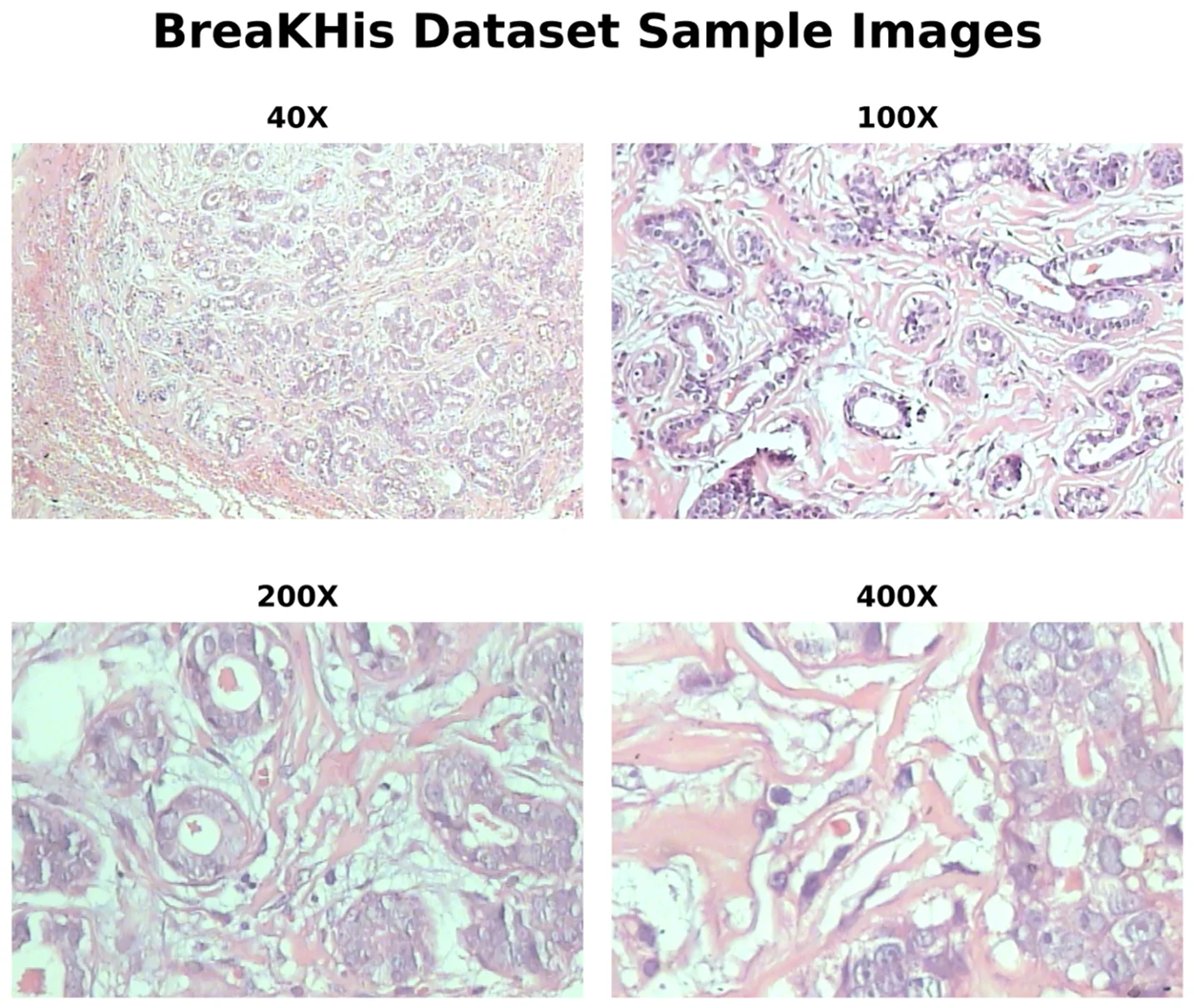
Histopathological image samples from BreakHis.

**Figure 2:**
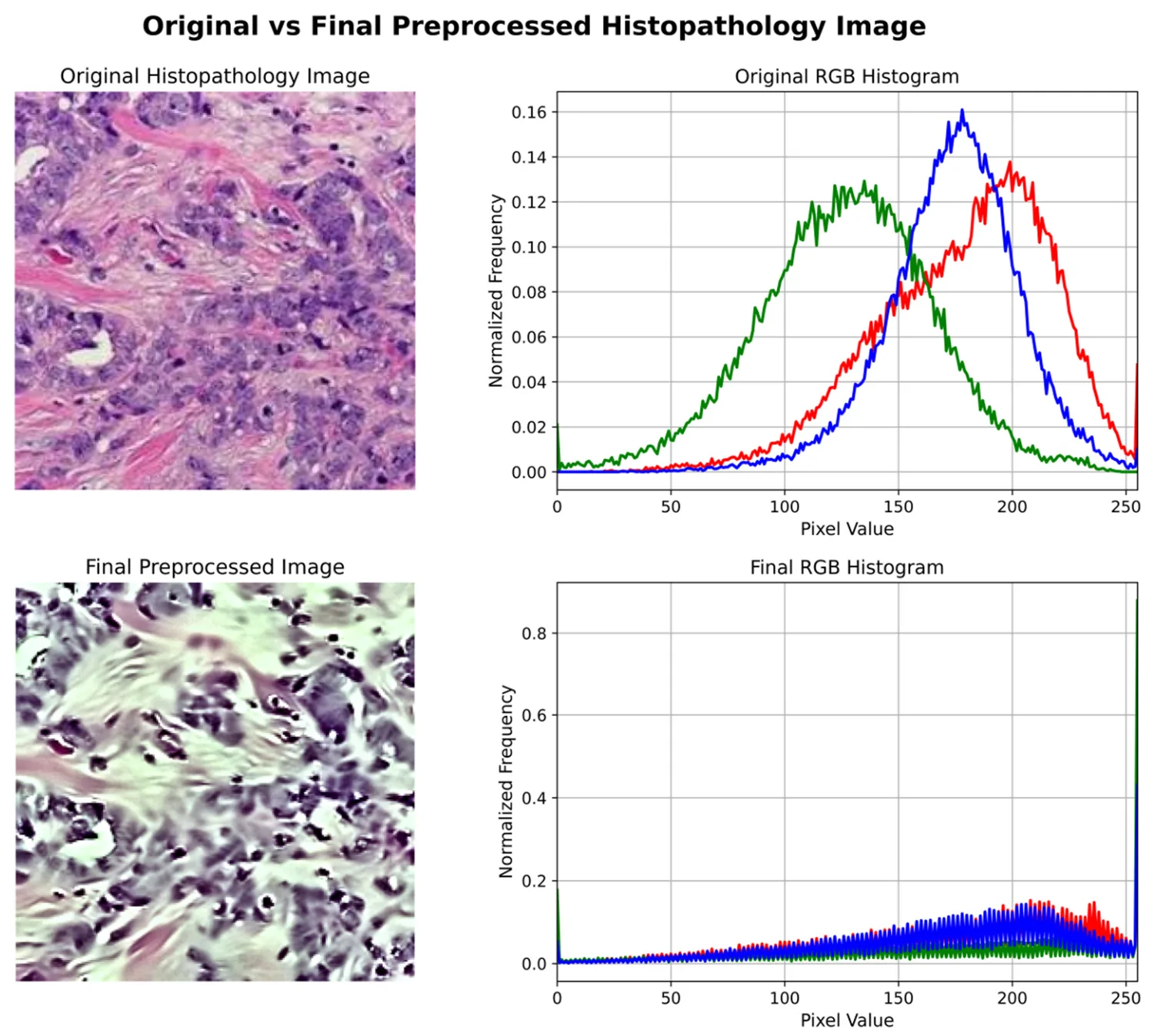
Histopathology Preprocessed Image Analysis. Images were acquired at 100× magnification.

**Figure 3:**
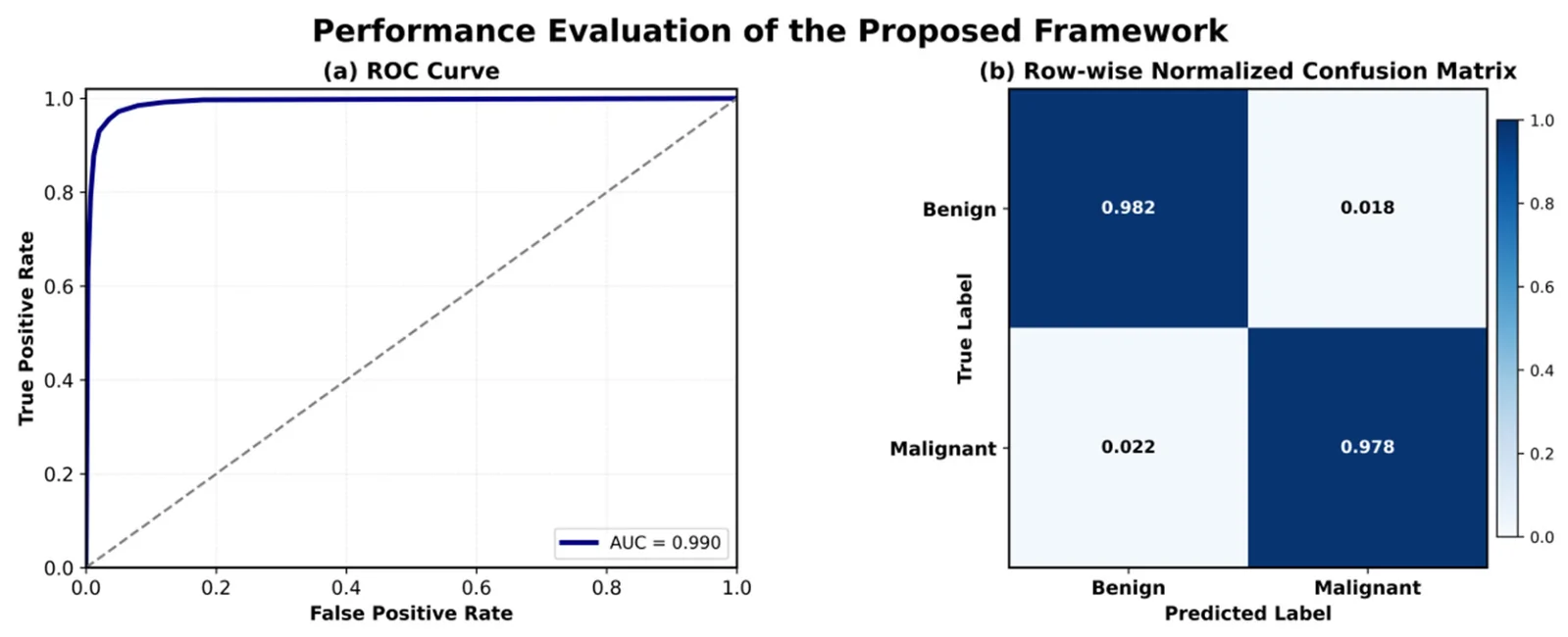
Performance analysis of the proposed framework. (**a**) ROC Curve; (**b**) Row-wise Normalized Confusion Matrix.

**Figure 4:**
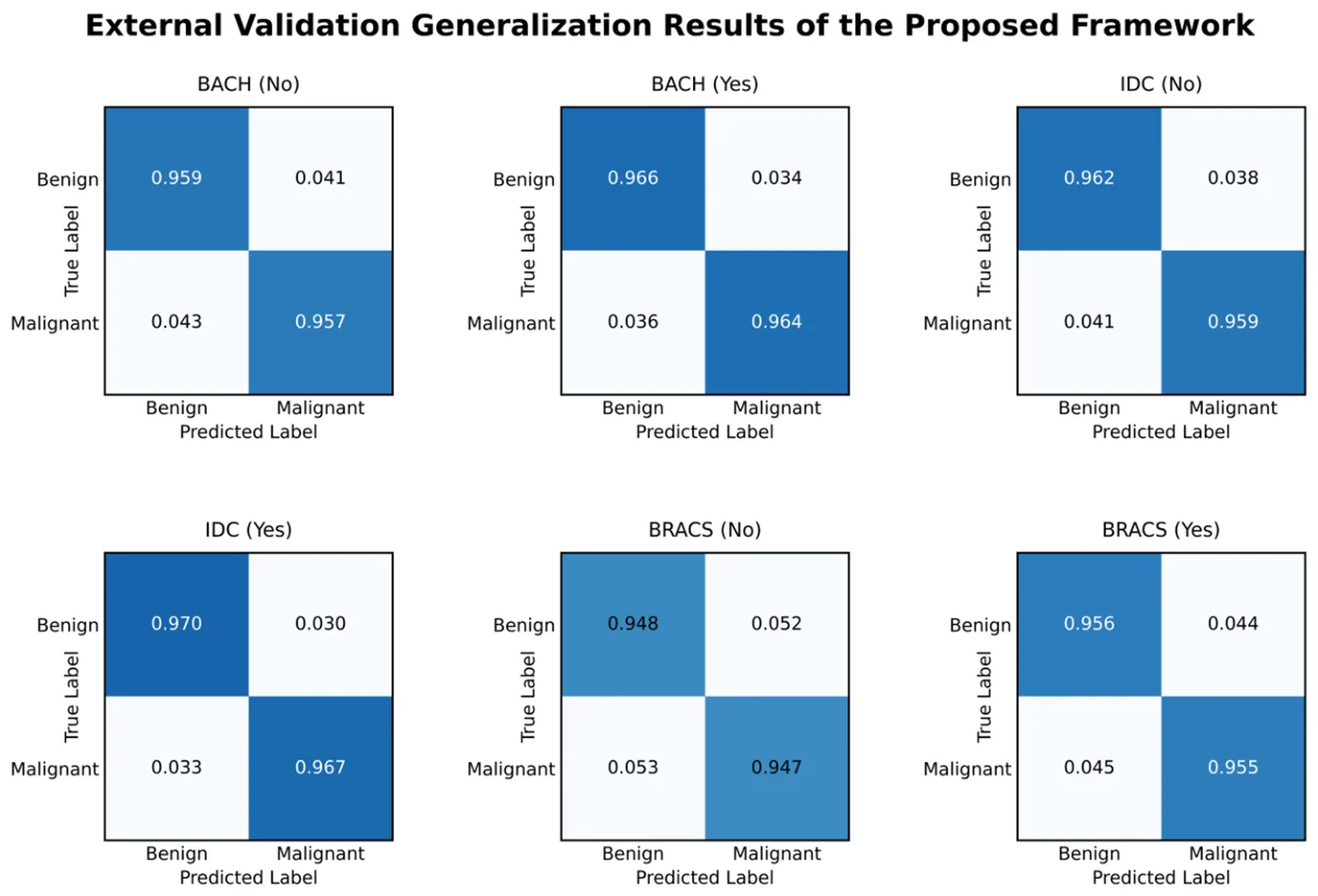
Comparative Row-Wise Confusion Matrix of Proposed Framework on External Datasets.

**Figure 5:**
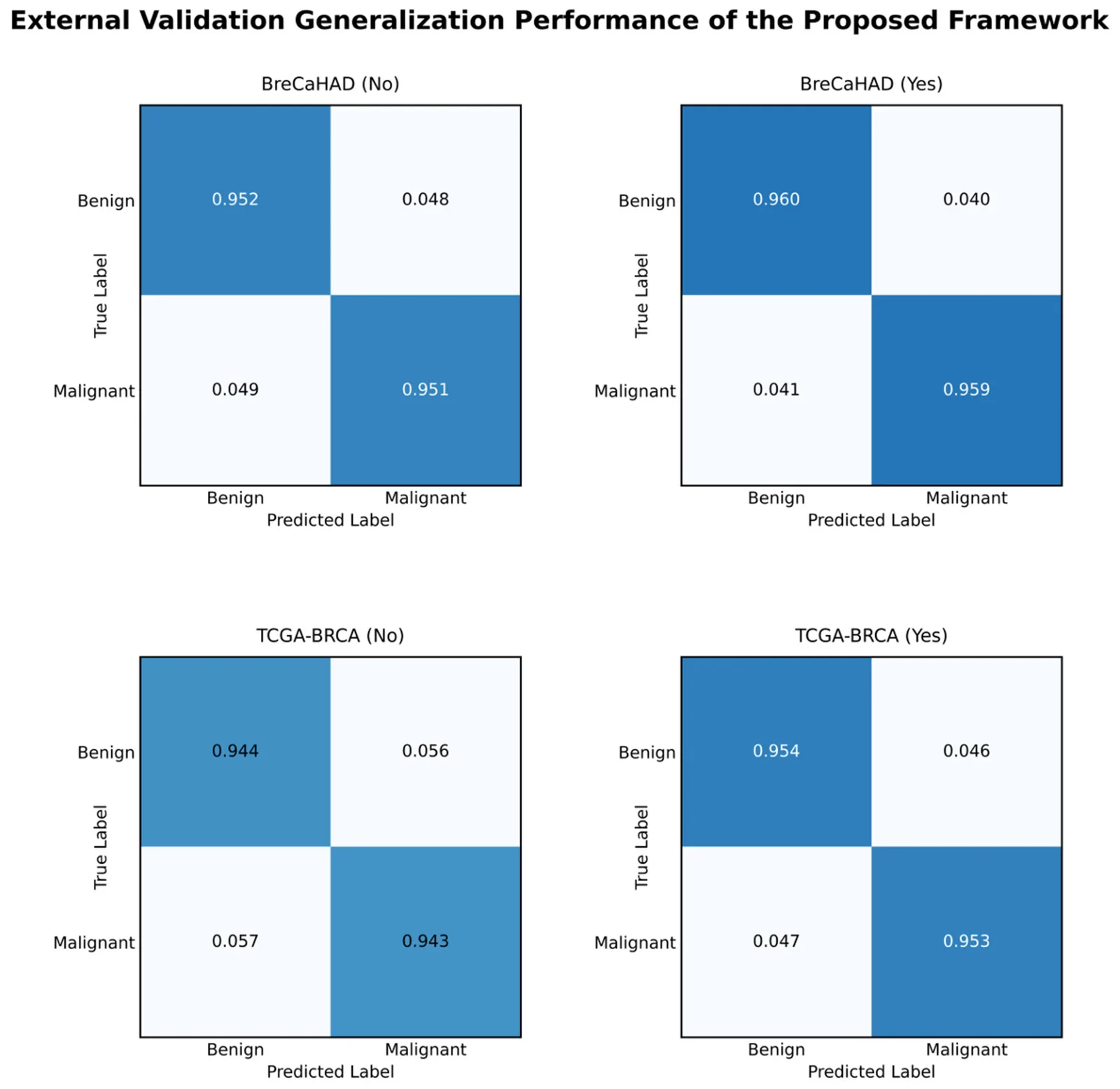
External validation generalization performance of the proposed framework.

**Figure 6:**
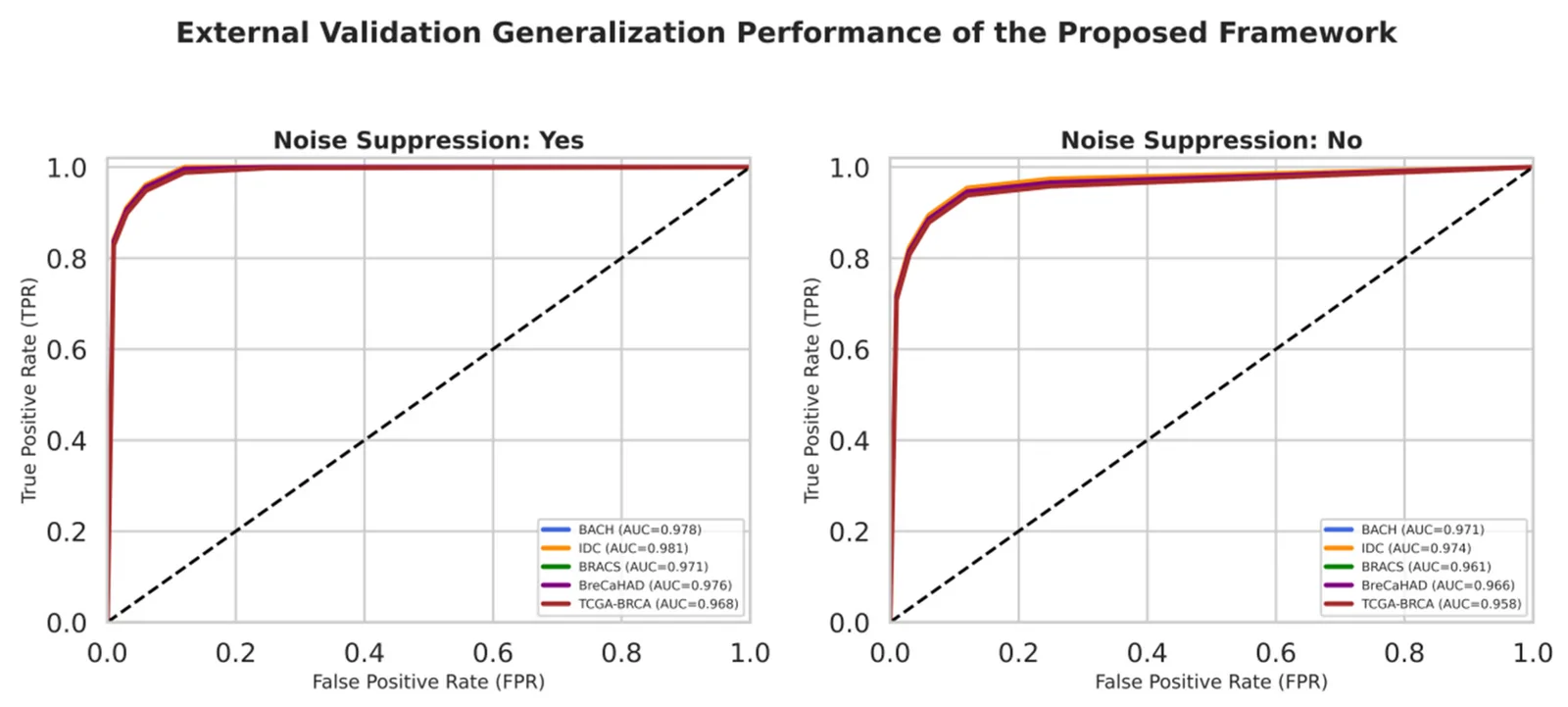
External validation ROC curves analysis of the proposed framework.

**Figure 7:**
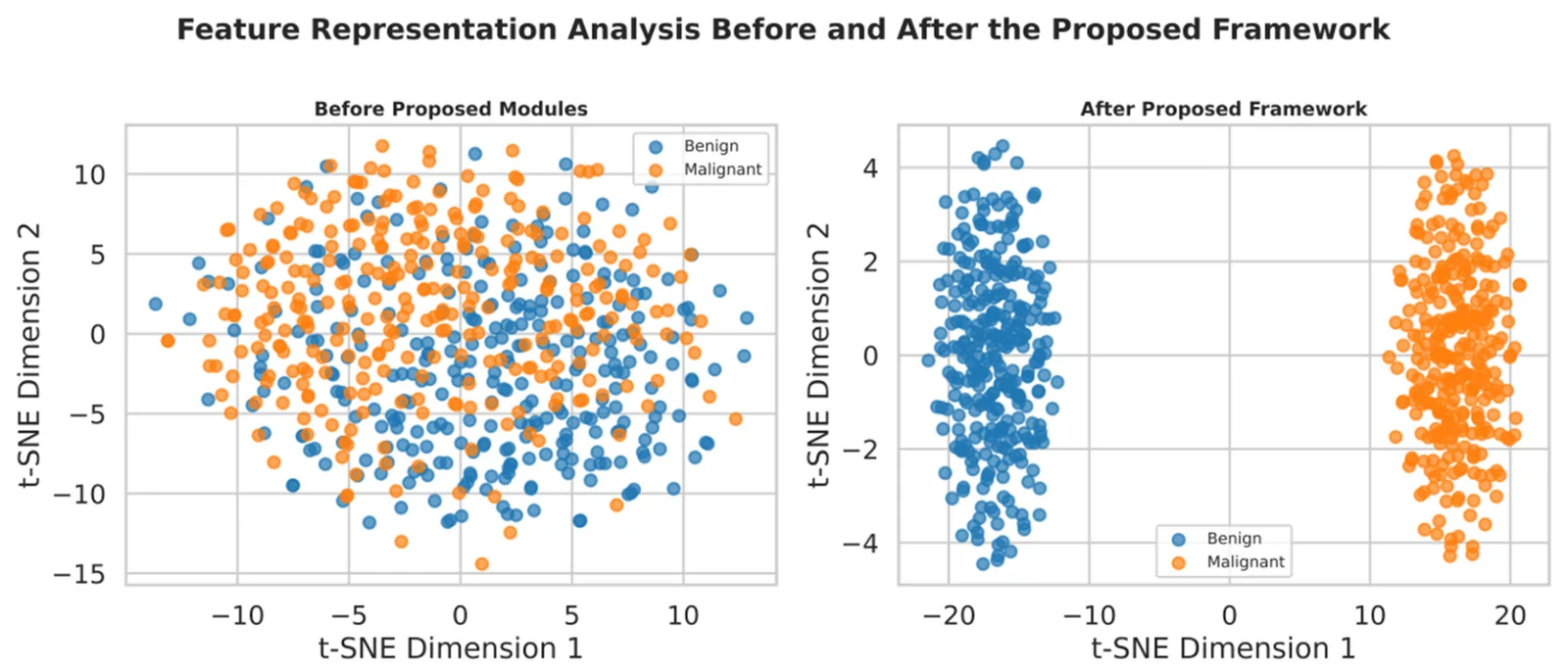
t-SNE visualization of feature representations before and after applying the proposed framework.

**Figure 8:**
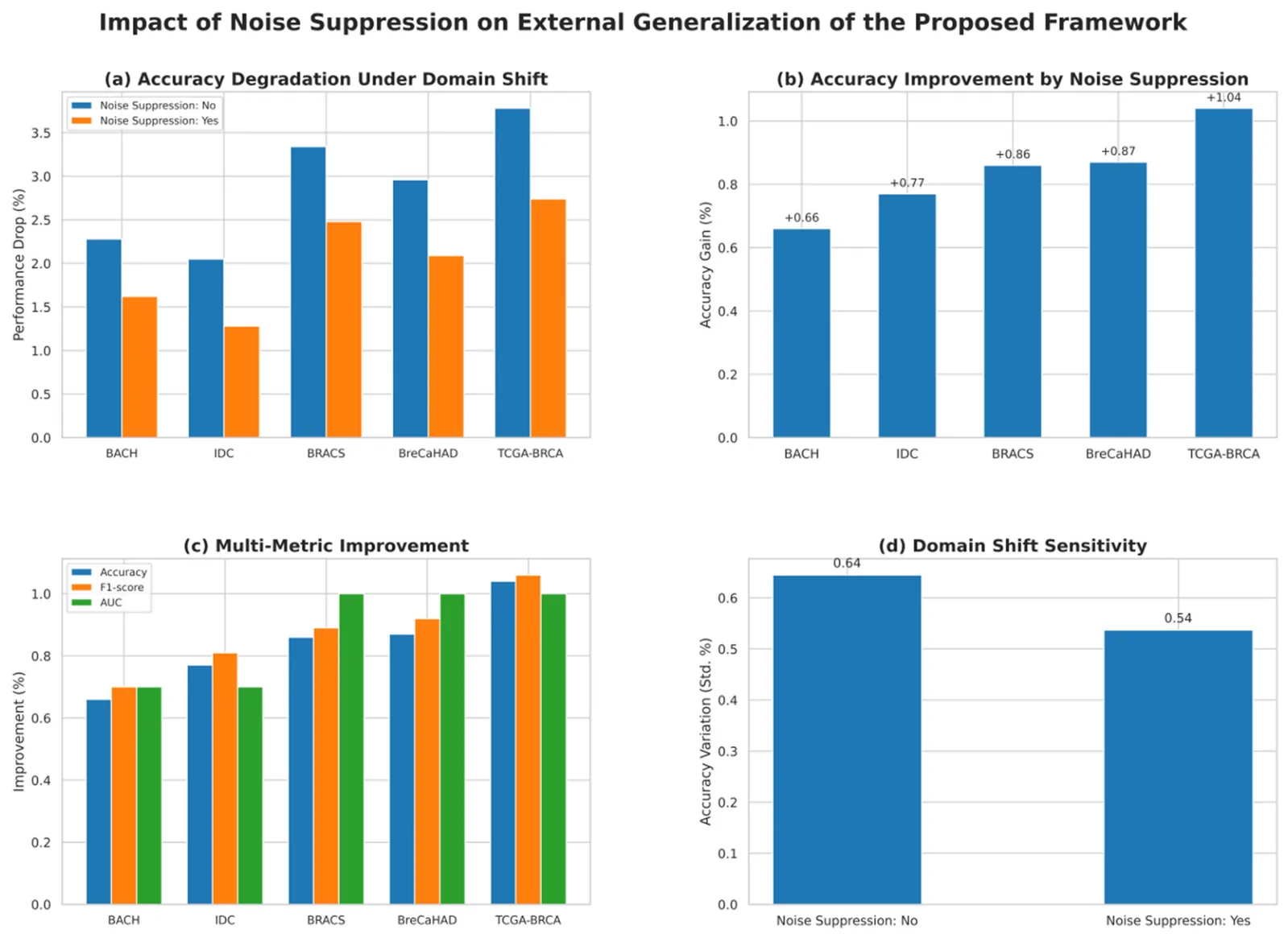
Comparative performance analysis of the proposed framework. (**a**) Accuracy Degradation Under Domain Shift; (**b**) Accuracy Improvement by Noise Suppression; (**c**) Multi-Metric Improvement; (**d**) Domain Shift Sensitivity.

**Figure 9:**
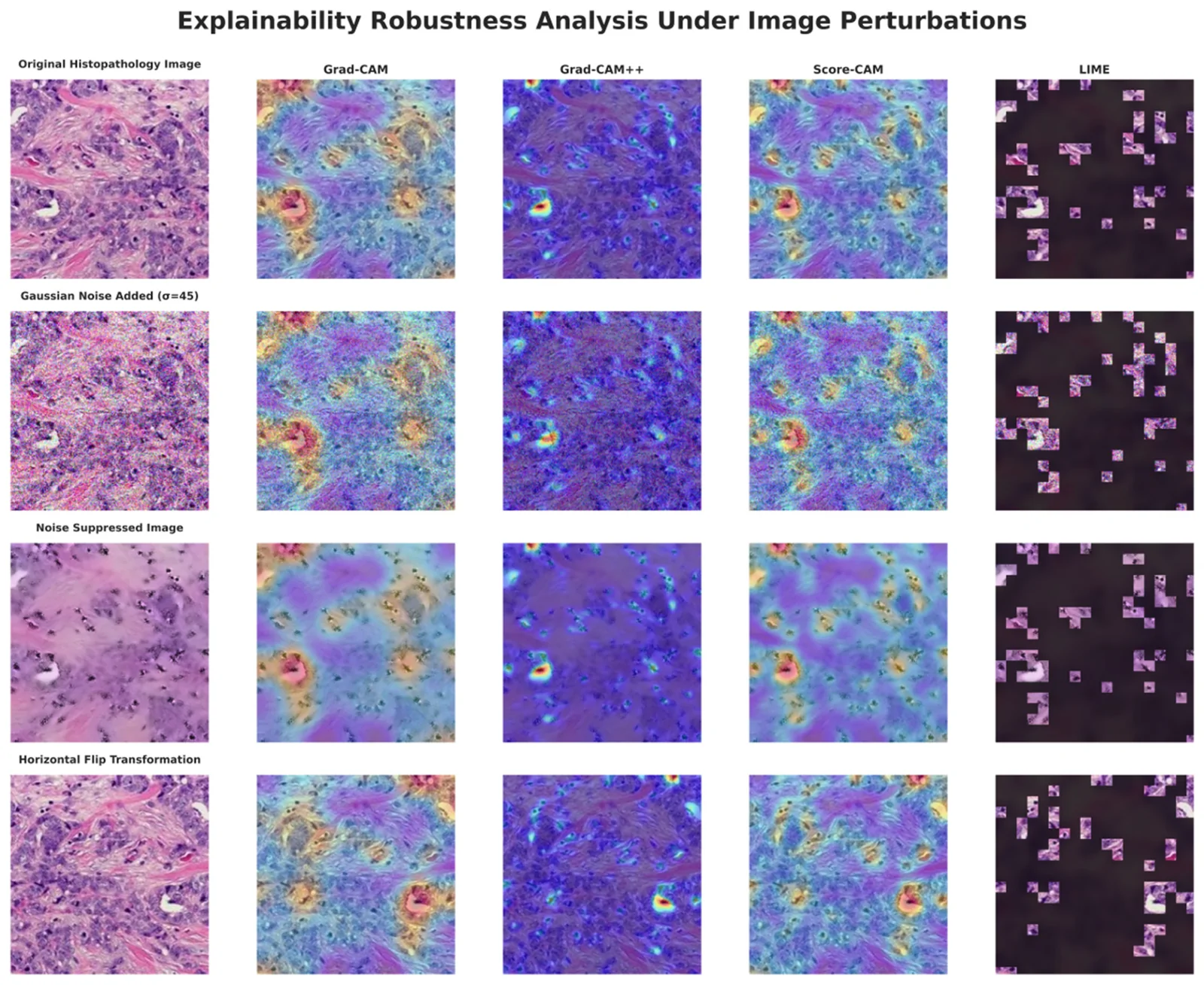
Explainable Analysis Using Different Methods.

**Figure 10:**
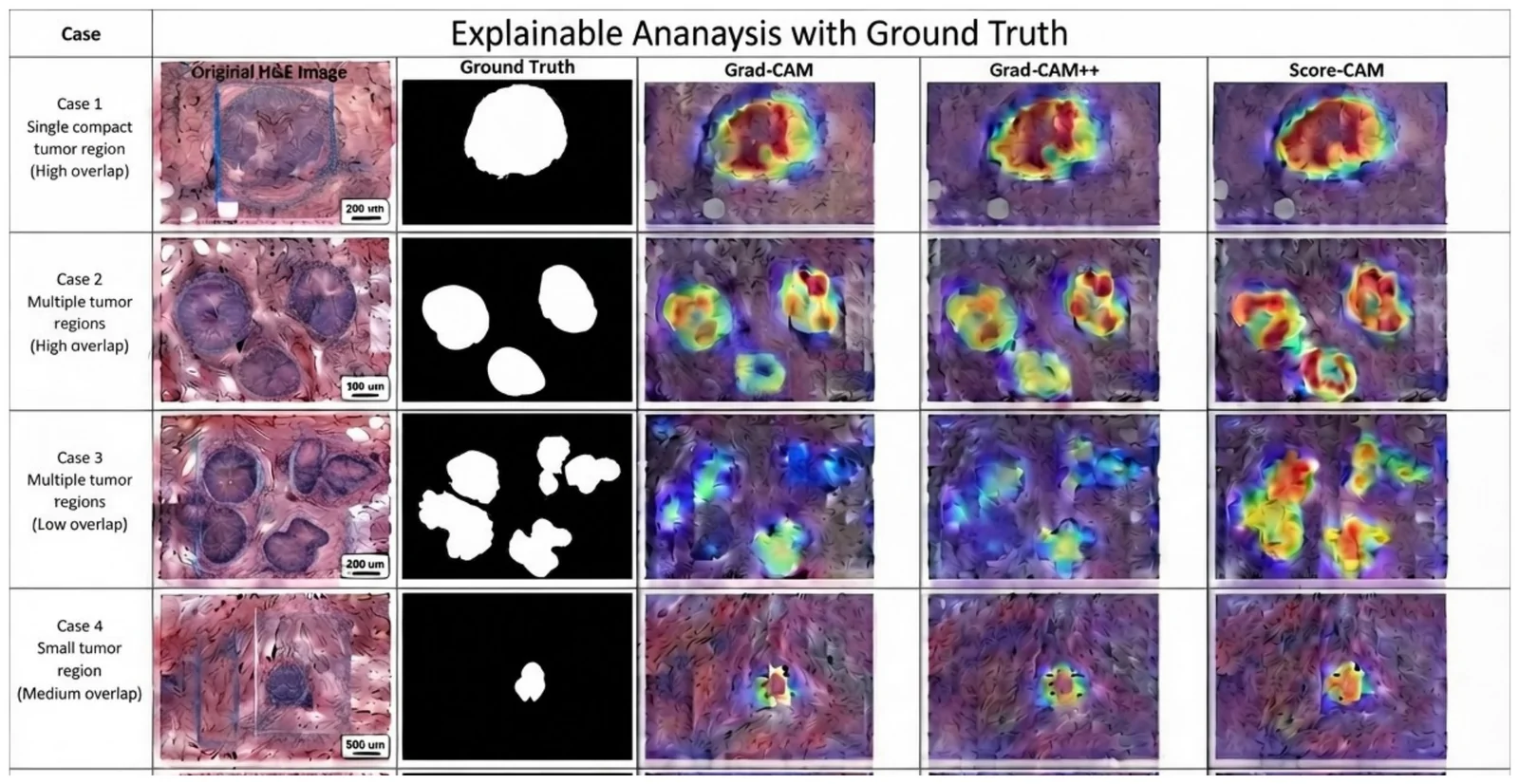
Explainable Analysis Using BCSS dataset.

**Table 1:** Summary of Limitations in Breast Cancer Histopathology Image Classification Models (2021–2026).

Study	Limitation 1	Limitation 2	Limitation 3	Key Contribution
[15]	No external validation	Performance degradation	Limited interpretability	Standard CNN-based classification
[28]	Overfitting	Dataset limitation	No XAI	CNN with transfer learning
[33]	No XAI	Insufficient generalization	Limited cross-magnification evaluation	Hybrid CNN–ViT for subtype classification
[6]	Dataset imbalance	No external validation	No stain normalization	Standard CNN with limited dataset
[11]	Dataset imbalance	No external validation	Limited interpretability	CNN for binary classification
[35]	No external validation	No XAI	Small dataset	ViT-based classification
[42]	Small dataset	Limited clinical validation	No cross-magnification test	CNN with patient-wise split
[44]	Dataset limitation	Overfitting	No XAI	Hybrid CNN–ViT with transfer learning

**Table 2:** Summary of Internal and External Validation Datasets Used in this Study.

Dataset	Role	Dataset Size	Patients	Image/Slide Type	Original Labels	Binary Labels Used in This Study
BreakHis	Internal evaluation	7909 images	82 patients	Histopathological images	Benign and Malignant histological subtypes	Benign and Malignant
BACH	External validation	400 images	NR	ROI images	Normal, Benign, In Situ Carcinoma, Invasive Carcinoma	Benign (Normal and Benign) and Malignant (In Situ Carcinoma and Invasive Carcinoma)
IDC	External validation	277,524 image patches	162 patients	ROI patches	IDC Negative, IDC Positive	Benign/non-IDC reference: IDC-negative; Malignant/IDC: IDC-positive
BRACS	External validation	4539 annotated regions from 547 WSIs	189 patients	Annotated/pre-extracted regions derived from WSIs	Normal (N), Pathological Benign (PB), UDH, FEA, ADH, DCIS, IC	Benign: N, PB, UDH; Malignant: DCIS, IC; FEA and ADH excluded
BreCaHAD	External validation	162 images	NR	Microscopic biopsy images	Mitosis, Apoptosis, Tumor Nuclei, Non-Tumor Nuclei, Tubule, Non-Tubule	External evaluation of the trained binary classification model on unseen breast cancer histopathology
TCGA-BRCA	External validation	1978 WSIs	1093 patients	WSI-derived/pre-extracted histopathological regions	Normal, Benign, Invasive Ductal Carcinoma (IDC), Invasive Lobular Carcinoma (ILC), Special Histologies, Mixed Histologies	Benign: Normal + Benign; Malignant: IDC + ILC + Special Histologies + Mixed Histologies

**Table 3:** Distribution of BreakHis Dataset for Internal Evaluation.

Dataset/Split	Patients	Benign Images	Malignant Images	Total Images	Percentage
Original	82	2480	5429	7909	100%
Training	57	1736	3800	5536	70% patients
Validation	12	372	814	1186	15% patients
Testing	13	372	815	1187	15% patients

**Table 4:** Summary of the Proposed Disease-Aware Intelligent Representation Learning Framework.

Component	Input	Main Operation	Mathematical Formulation	Output	Role
VGG16 CNN Encoder	Histo image X	Hierarchical convolution-based feature extraction	$Z_{c}=f_{CNN}(X;\theta_{c})$	CNN feature representation $Z_{c}$	Extracts local morphological and textural features
Swin Transformer Encoder	Histopathological image X	Patch-based self-attention feature learning	$Z_{v}=f_{ViT}(X;\theta_{v})$	$Z_{v}$	Captures global contextual dependencies
FAM	$Z_{c}$, $Z_{v}$	Feature projection and latent space alignment	$P_{c}=W_{c}Z_{c}+b_{c}$, $P_{v}=W_{v}Z_{v}+b_{v}$, $Z_{a}=P_{c}+P_{v}$	Aligned representation ($Z_{a}$)	Harmonizes heterogeneous CNN and Transformer features into a unified latent space.
DAFSM	$Z_{a}$	Disease relevance gating and feature disentanglement	$G_{d}=\sigma (W_{g}Z_{a}+b_{g})$, $Z_{d}=G_{d}⊙Z_{a}$, $Z_{n}={(1-G}_{d})⊙Z_{a}$	Disease representation $Z_{d}$, nuisance representation $Z_{n}$	Separates diagnostic information from non-disease variations and suppresses irrelevant features
DEAM	$Z_{d}$	Adaptive attention-based feature refinement	$A=Softmax(W_{a}Z_{d})$, $Z_{e}=A⊙Z_{d}$, $Z_{enh}=Z_{d}+Z_{e}$	Enhanced disease representation $Z_{enh}$	highlights discriminative pathological evidence while preserving original disease features
AIDM	$Z_{enh}$	Confidence estimation and adaptive classification	$C=\sigma (W_{c}Z_{enh}+b_{c})$, $Z_{c}=C⊙Z_{enh}$, $\hat{y}=Softmax(W_{p}Z_{c}+b_{p})$	Final prediction $\hat{y}$	Produces confidence-guided diagnostic decisions with adaptive feature weighting

**Table 5:** Experimental Design and Training Setup.

Category	Parameter(s)	Configuration
Dataset Configuration	Dataset, Classification Task, External validation, Image Resolution	BreakHis dataset for internal evaluation; BACH, IDC, BRACS, BreCaHAD, and TCGA-BRCA for external validation after binary mapping; Binary classification (Benign/Malignant); Input image size of 224 × 224 × 3 RGB.
Data Preparation	Data Split, Cross-Validation, Preprocessing and Augmentation	Patient-wise 70-15-15 train-validation-test split with five-fold stratified cross-validation; resize = 224 × 224, stain normalization = Macenko method, pixel normalization = [0, 1], NLM Denoising = Patch size = 7, Search window = 11, h = 10, Augmentation = Rotation ±20°, Flip *p* = 0.5, Zoom 0.8–1.2, Translation ±10%, Shear ±10°, Brightness ±20%, Contrast ±20%, Class, *p* = 0.3,Balancing = Training set only, Validation/Test = Resize + normalization only
Baseline Backbone Selection	CNN and Vision Transformer Models	ResNet50 and VGG16 are used as CNN baselines, while ViT-B/16 and Swin Transformer are employed as Vision Transformer baselines. The highest-performing CNN and Vision Transformer are selected according to validation Accuracy, F1-score, and AUC.
Feature Representation Learning	Feature Dimensions and Projection	CNN feature representation (2048-D) and Vision Transformer embedding (768-D) are projected into a unified 512-dimensional latent space for heterogeneous feature representation learning.
Cross-Representation Feature Alignment Module (FAM)	Alignment Strategy	Linear projection, feature transformation, latent-space alignment, and element-wise feature fusion are performed to minimize representation discrepancy between CNN and Vision Transformer features.
DAFSM	Disease Feature Disentanglement	Fully connected gating network with Sigmoid activation (Input = 1024, Hidden = 512) separates disease-specific and nuisance representations using $F_{d}$ = G⊙$F,\;F_{n}==(1-G)⊙F$
DEAM	Attention Refinement	Softmax-based disease evidence attention with residual refinement adaptively emphasizes diagnostically important pathological regions while suppressing irrelevant information.
Model Optimization	Training Configuration	AdamW optimizer, learning rate 1 × 10^−4^, weight decay 1 × 10^−5^, ReduceLROnPlateau scheduler, batch size 32, 100 epochs, early stopping (patience = 15), dropout 0.30, random seed 42, ReLU/GELU/Sigmoid/Softmax activation, Batch Normalization and Layer Normalization.
Objective Function	Loss Formulation	Cross-entropy classification loss combined with Disease Separation Loss using $L_{total}=L_{cls}+{\lambda L}_{sep},where\;\lambda =0.1$
Performance Evaluation	Evaluation Metrics and Explainability	Accuracy, Precision, Recall, Specificity, F1-score, AUC, and Cohen’s Kappa are used for quantitative evaluation, while Grad-CAM, Grad-CAM++, and Score-CAM provide visual interpretability.

**Table 6:** Baseline Performance with ImageNet Pre-training (Repeated 5-Fold CV).

Model Category	Model	Accuracy (%)	Precision (%)	Recall (%)	F1-Score (%)	AUC	Std
CNN	ResNet50	92.84	92.31	92.08	92.19	0.956	±0.28
CNN	VGG16 (Selected)	93.40	93.18	93.02	93.10	0.965	±0.24
ViT	ViT-B/16	94.21	93.89	93.74	93.81	0.971	±0.22
ViT	Swin Transformer (Selected)	94.70	94.48	94.31	94.39	0.978	±0.19

**Table 7:** Ablation Study of the Proposed Components on the Internal BreakHis Dataset.

Configuration	FAM	DAFSM	DEAM	AIDM	Noise Suppression	Accuracy (%)	Precision (%)	Recall (%)	F1 (%)	Kappa	MCC	AUC
Baseline (FC + Softmax)	No	No	No	No	No	96.95 ± 0.12	96.62 ± 0.14	96.91 ± 0.13	96.7 ± 0.13	0.938 ± 0.006	0.93 ± 0.006	0.97 ± 0.004
+FAM	Yes	No	No	No	Yes	97.23 ± 0.11	97.54 ± 0.12	97.81 ± 0.11	97.6 ± 0.11	0.953 ± 0.005	0.95 ± 0.005	0.98 ± 0.003
+DAFSM	Yes	Yes	No	No	Yes	97.57 ± 0.09	97.70 ± 0.10	97.88 ± 0.09	97.7 ± 0.09	0.956 ± 0.004	0.95 ± 0.004	0.98 ± 0.003
FAM + DEAM	Yes	No	Yes	No	Yes	97.74 ± 0.09	97.78 ± 0.10	97.91 ± 0.09	97.8 ± 0.09	0.957 ± 0.004	0.95 ± 0.004	0.98 ± 0.003
FAM + AIDM	Yes	No	No	Yes	Yes	97.68 ± 0.10	97.73 ± 0.11	97.86 ± 0.10	97.7 ± 0.10	0.956 ± 0.004	0.95 ± 0.004	0.98 ± 0.003
+DEAM	Yes	Yes	Yes	No	Yes	97.93 ± 0.07	97.82 ± 0.09	97.95 ± 0.08	97.8 ± 0.08	0.959 ± 0.004	0.96 ± 0.004	0.98 ± 0.003
FAM + DAFSM + AIDM	Yes	Yes	No	Yes	Yes	98.01 ± 0.06	97.88 ± 0.08	98.01 ± 0.07	97.9 ± 0.07	0.961 ± 0.003	0.96 ± 0.003	0.99 ± 0.002
FAM + DEAM + AIDM	Yes	No	Yes	Yes	Yes	97.96 ± 0.07	97.84 ± 0.08	97.96 ± 0.08	97.9 ± 0.07	0.960 ± 0.004	0.96 ± 0.004	0.98 ± 0.003
+AIDM/Full DAIRLM	Yes	Yes	Yes	Yes	Yes	98.10 ± 0.05	97.92 ± 0.06	98.03 ± 0.05	97.9 ± 0.05	0.962 ± 0.003	0.96 ± 0.003	0.99 ± 0.002

**Table 8:** External Validation Ablation Analysis on Multiple Breast Histopathology Datasets.

External Dataset	Noise Suppression	Accuracy (%)	Sensitivity (%)	Specificity (%)	F1-Score (%)	Kappa	MCC	AUC
BACH	No	95.82	95.70	95.90	95.61	0.916	0.918	0.971
BACH	Yes	96.48	96.40	96.60	96.31	0.929	0.931	0.978
IDC	No	96.05	95.90	96.20	95.84	0.921	0.923	0.974
IDC	Yes	96.82	96.70	97.00	96.65	0.936	0.938	0.981
BRACS	No	94.76	94.70	94.80	94.52	0.895	0.897	0.961
BRACS	Yes	95.62	95.50	95.60	95.41	0.912	0.914	0.971
BreCaHAD	No	95.14	95.10	95.20	94.92	0.903	0.905	0.966
BreCaHAD	Yes	96.01	95.90	96.00	95.84	0.920	0.922	0.976
TCGA-BRCA	No	94.32	94.30	94.40	94.08	0.886	0.888	0.958
TCGA-BRCA	Yes	95.36	95.30	95.40	95.14	0.907	0.909	0.968

**Table 9:** Class-wise Performance on External Datasets.

External Dataset	Class	Sensitivity (%)	Specificity (%)	95% CI
BACH	Benign	96.60	96.40	94.19–97.89
	Malignant	96.40	96.60	94.19–97.89
IDC	Benign	97.00	96.70	96.75–96.88
	Malignant	96.70	97.00	96.75–96.88
BRACS	Benign	95.60	95.50	94.99–96.18
	Malignant	95.50	95.60	94.99–96.18
BreCaHAD	Benign	96.00	95.90	91.78–98.11
	Malignant	95.90	96.00	91.78–98.11
TCGA-BRCA	Benign	95.40	95.30	94.34–96.20
	Malignant	95.30	95.40	94.34–96.20

**Table 10:** Disease–Nuisance Representation Probe Analysis.

Representation	Disease Probe Accuracy (%) ↑	Disease Probe F1 (%) ↑	Domain Probe Accuracy (%) ↓	Domain Probe F1 (%) ↓
Fused Representation (F)	96.84	96.72	76.35	75.81
Disease Representation (Zd)	97.91	97.84	41.27	39.86
Nuisance Representation (Zn)	63.48	60.92	93.76	93.21

↑: higher/better performance; ↓: lower/worse performance.

**Table 11:** Comprehensive Statistical Significance Analysis of the Proposed Framework against Baseline Models.

Statistical Test	Comparison	N (Folds)	Test Statistic	*p*-Value	Effect Size	95% CI of Accuracy Difference
Paired *t*-test	Proposed vs. ResNet50	5	t = 53.60	<0.001	Cohen’s d = 23.97	5.01–5.51
Paired *t*-test	Proposed vs. VGG16	5	t = 48.32	<0.001	Cohen’s d = 21.62	4.43–4.97
Paired *t*-test	Proposed vs. ViT-B/16	5	t = 36.71	<0.001	Cohen’s d = 16.42	3.65–4.13
Paired *t*-test	Proposed vs. Swin Transformer	5	t = 38.33	<0.001	Cohen’s d = 17.14	3.20–3.60
Friedman test	ResNet50 vs. VGG16 vs. ViT-B/16 vs. Swin vs. Proposed	5	χ^2^ = 20.00	0.0005	Kendall’s W = 1.00	—

**Table 12:** Overall Quantitative Evaluation of XAI Methods on BCSS.

XAI Method	Dice Score ↑	IoU ↑	Precision ↑	Recall ↑	Localization Accuracy ↑
Grad-CAM	0.736 ± 0.019	0.586 ± 0.018	0.772 ± 0.021	0.751 ± 0.020	0.810 ± 0.016
Grad-CAM++	0.788 ± 0.018	0.652 ± 0.021	0.813 ± 0.019	0.796 ± 0.018	0.850 ± 0.015
Score-CAM	0.768 ± 0.018	0.622 ± 0.022	0.798 ± 0.020	0.777 ± 0.018	0.830 ± 0.015

↑: higher/better performance.

**Table 13:** Comparison of the Proposed Framework with Conventional CNN–Transformer Hybrid Architectures.

Aspect	Conventional CNN–ViT Hybrid Methods	Proposed Framework
CNN–ViT integration	Direct concatenation, addition, or generic fusion	FAM-based latent-space alignment before fusion
Feature representation	Unified fused representation	Fused representation is explicitly decomposed into $Z_{d}$ and $Z_{n}$
Disease/nuisance handling	Usually not explicitly modeled	DAFSM encourages disease-oriented and complementary feature separation
Separation constraint	Generally absent	Orthogonality-based $L_{Sep}$
Attention location	Generic fused/intermediate features	DEAM specifically refines $Z_{d}$
Decision stage	Conventional classifier/Softmax	AIDM adaptively reweights refined disease features before classification
Overall pathway	Fusion → classification	Alignment → decomposition → disease-evidence refinement → adaptive decision

**Table 14:** Summary of Performance Comparison and Limitations in Breast Cancer Image Classification Models.

Author	Dataset	Method	Accuracy (%)	F1 (%)
[27]	Mammogram images	CNNs-ViT	90.1	0.90
[7]	Clinical Dataset	ML and CNN	93.0	0.92
[23]	DDSM	CNNs-ViT	99.62	0.9982
[41]	BCW	(CB + LGBM + ET) + CNN	0.9772	0.9819
[15]	Mammograms	CNNs	0.748	0.775
[17]	MIAS	DL Models	93.0	94.0
Proposed work	BreakHis and External Datasets	Intelligent Hybrid	98.1	97.7

## Data Availability

This study has been conducted using six publicly available datasets. Links for downloading these datasets are provided below. BreakHis: https://web.inf.ufpr.br/vri/databases/breast-cancer-histopathological-database-breakhis/ (accessed on 7 September 2026); BACH: https://iciar2018-challenge.grand-challenge.org/ (accessed on 7 September 2026); IDC: https://www.kaggle.com/datasets/paultimothymooney/breast-histopathology-images (accessed on 7 September 2026); BRACS: https://www.bracs.icar.cnr.it/ (accessed on 7 September 2026); TCGA-BRCA: https://portal.gdc.cancer.gov/projects/TCGA-BRCA (accessed on 7 September 2026); BCSS: https://bcsegmentation.grand-challenge.org/ (accessed on 7 September 2026).

## Abbreviations

The following abbreviations are used in this manuscript:

CNN	Convolutional Neural Network
ViT	Vision Transformer
MLP	Multilayer Perceptron
DL	Deep Learning
GAP	Global Average Pooling
Grad-CAM	Gradient-weighted Class Activation Mapping
DC	Ductal Carcinoma
ML	Machine Learning
ROC	Receiver Operating Characteristic Curve
AUC	Area Under the Curve
Std	Standard Deviation
RAM	Random Access Memory
MIL	Multiple Instance Learning
